# The risk for complications and reoperations with the use of mega prostheses in bone reconstructions

**DOI:** 10.1186/s13018-021-02749-z

**Published:** 2021-10-14

**Authors:** Christina Berger, Sofia Larsson, Peter Bergh, Helena Brisby, David Wennergren

**Affiliations:** 1 Institute of Clinical Sciences, Sahlgrenska Academy, University of Gothenburg, Gothenburg, Sweden; 2 Department of Orthopaedics, Sahlgrenska University Hospital, Bruna Stråket 11B, 413 45 Gothenburg, Sweden

**Keywords:** Mega prostheses, Orthopaedic oncology, Bone reconstruction, Complications, Bone tumours

## Abstract

**Background:**

Despite a relatively high risk for complications and reoperations, mega prostheses are considered a useful method for reconstruction of bone defects after tumour resections. The total number of reoperations has not previously been described, and little is known about the complication rate of mega prostheses used for other indications than primary bone tumours.

**Questions/purposes:**

The current retrospective observational study aimed to describe the patient population treated with mega prostheses at Sahlgrenska University Hospital, Sweden, during 14 consecutive years, reports the complications leading to reoperation and the number and type of reoperations for different kinds of complications, and reports on implant survival.

**Methods:**

All patients treated with a mega prosthesis, regardless of surgical indication and anatomical location, at Sahlgrenska University Hospital during the period 2006–2019 were identified. The medical records for all patients were reviewed. Data regarding age, sex, diagnosis, site of disease, bone resection length, chemotherapeutical treatment and postoperative complications including infections and oncological outcome, were collected and evaluated.

**Results:**

One hundred and fourteen patients treated with 116 mega prostheses were included in the study. The predominant indication for primary surgery with a mega prosthesis was sarcoma of either bone or soft tissue (53.5% of the patients). In total 51 prostheses (44%) did not require any reoperation after the primary surgery. The most common reason for reoperation was infection (22%) followed by soft tissue failure (13%). The risk for prosthetic infection was significantly higher in the group of patients operated due to sarcoma compared with all other indications for surgery regardless of surgical site (*p* = 0.004).

**Conclusion:**

The study reveals a total reoperation rate of 56% after reconstructive surgery using mega prostheses. Despite the high reoperation rates, at the end of the study period, 83% of the patients had still a functioning prosthesis. Therefore, the use of mega prostheses can be considered a reliable method for reconstruction of large bone defects in selected patients.

**Level of Evidence:**

Level IV, therapeutic study.

## Background

During the last 4 decades the use of mega prostheses, also known as tumour endoprostheses, has radically changed the treatment options for patients with malignant primary bone tumours. Mega prostheses are modular endoprostheses, consisting of components that can be assembled in different combinations to fit the specific skeletal defect and allow limb sparing. Today it is considered to be a safe and convenient reconstructive method for upper and lower extremities after tumour resections, with similar failure rates as with reconstruction with allograft [1, 2]. A recent systematic review of the literature showed no significant differences in oncological outcome between amputation and mega prosthesis reconstructions [3]. Despite the need for wide surgical margins in order for malignant primary bone tumours to ensure complete removal, the use of mega prostheses, together with refined imaging technology and improved oncological treatment, has changed the preferred surgical treatment from amputation to limb-sparing surgery at many centres [4].

One major advantage with mega prosthesis is the allowance of immediate weight-bearing postoperatively, leading to a faster rehabilitation compared with other methods. However, the surgery is often performed in young patients, some still growing, with a long remaining life expectancy and high demands on function which require excellent implant quality.

Aside from reconstructions after tumour surgery, the indications for mega prostheses have been expanded to include also other medical conditions, such as comminute fractures involving the knee joint and revision surgery after conventional knee/hip endoprosthesis failures [5]. However, there are factors connected to mega prostheses that result in a relatively high risk for complications. An overall complication rate between 15 and 45%, including severe complications such as deep infections, soft tissue failures, mechanical failures and aseptic loosening, has been reported [6–10]. This is far higher than reported complication rates after conventional total arthroplasty of hip, knee and shoulder [11]. Furthermore, mega prostheses are associated with higher risks for complications than revision stems; Perticarini et al. [12] showed promising survivorship in cases of complex femoral bone defect. Not only do complications, such as those listed above, result in reoperations, higher medical costs and increased patient suffering, they may also eventually lead to a joint resection arthrodesis, or an amputation as final outcome.

The first aim of this study was to describe the patient population treated with mega prostheses at Sahlgrenska University Hospital during 14 consecutive years. The second aim was to evaluate the complication rate, according to infections, soft tissue failure, mechanical failure, aseptic loosening and tumour progression, and any required reoperations. The third aim was to investigate overall implant survival.

## Methods

### Data

All patients treated with a mega prosthesis, regardless of indication for surgery and anatomical location, at Sahlgrenska University Hospital during the period from January 2006 to May 2019 were identified using the hospital’s operation planning systems and included in the study. All primary surgeries, follow-up and eventual additional surgeries were performed by four surgeons, all of whom were subspecialists in orthopaedic oncology with additional extensive trauma surgery experience.

Background data regarding age, sex, diagnosis, site of disease, bone resection length, chemotherapeutical treatment, postoperative complications including infections and oncological outcome were collected from patient medical records. Furthermore, data regarding neoadjuvant or adjuvant chemotherapy treatment, were collected for all patients with primary bone tumours.

To examine the possible relationship between immunosuppressive effect in close proximity to index surgery and complication rate, data regarding systemic chemotherapy treatment approximately 4 weeks before or after index surgery were collected for patients with metastatic disease.

Data regarding complications after surgery with a mega prosthesis were collected from the medical records. Complications were classified according to the Henderson five type classification [13]: Type 1, soft tissue failures (skin necrosis, flap insufficiency and stiffness/contracture of the reconstructed joint were defined as soft tissue failure); Type 2, aseptic loosening failures; Type 3, structural failures, i.e. fractures of prosthetic components; Type 4, infection; and Type 5, tumour prolapse/progression. Data regarding all complications that led to reoperation/revision of the prosthesis were collected as well as data regarding all reoperations performed (i.e. all reoperations regardless of type of surgery). A revision was defined as a reoperation where any component of the prosthesis was replaced. The number of reoperations, reason, type of reoperation and time from primary surgery to first reoperation for each patient were recorded together with data regarding status, including oncologic status, at latest follow-up. The data were reported according to the STROBE guidelines [14].

### Mega prostheses

The mega prosthesis primarily used was the Modular Universal Tumor and Revision System, MUTARS™ (Implant Cast, Germany). Both uncemented and cemented stems were used depending on the prerequisites of the bone, site and patient. Most hip replacements were hemiarthroplasties with a dual-mobility cup. Shoulder replacements were either unipolar or reversed total replacements. Rotating hinged knee was used in the total knee replacements. The design of the hinge was changed during the study period; a metal-PEEK™ (polyether ether ketone) coupling was used 2006–2014 and was then changed to the metal-on-metal (MoM) coupling used today.

In smaller, still growing, children, an expandable prosthesis allowing lengthening was used (MUTARS Expand™). Parents, or the patients themselves performed the lengthening procedure at home, with a transcutaneous microwave induction transducer activating a motor-driven telescopic device located inside the prosthesis. No further surgical procedures for lengthening were needed. However, after completion of the lengthening, the motor-driven prosthesis needed to be exchanged to a definitive prosthesis. For large resections of the pelvis, an ice-cone pedestal acetabular replacement according to the LUMIC™ (Implant Cast, Germany) system in combination with a standard Lubinus™ (Link™) femur stem, was used.

### Statistical methods

Descriptive statistics is given for the study population, with percentages and mean together with range presented where relevant.

A Kaplan–Meier analysis was performed to calculate the implant survival. In the Kaplan–Meier analysis revision, extraction of prosthesis and amputation due to complication were set as events of interest. Patients who died, were amputated or revised due to tumour progression during the study period were censored.

Multivariable regression analysis was performed for calculation of increased risk of infection or other complications. The factors evaluated were anatomic site, length of resection, diagnosis, chemotherapy or radiation in connection with the surgery.

All statistical analyses were performed using IBM SPSS version 27.

## Results

### Demographics

A total of 115 patients (55 females (48.2%)) operated with 117 prostheses during the study period were identified. One patient was lost to follow-up due to emigration 1 year after surgery and excluded from the study. Two patients were provided with two prostheses in two different extremities. Thus 114 patients treated with 116 prostheses were evaluated. The mean age at primary surgery was 53.4 years (range 7.1–88.1 years), and the mean follow-up time was 7.6 years (range 1.3–13.3 years) (Table 1). At the end of the study, 56 (49%) patients were alive and 58 (51%) patients had died. All patients who died during the study period were followed until their death.

**Table 1:** Number of patients, mean age at primary surgery according to indication for surgery and surgical site

	Total number of patients	Mean age at primary surgery (range)	Female (%)	Total number of prostheses	Proximal humerus	Total humerus	Intercalary humerus	Distal humerus	Pelvis	Proximal femur	Total femur	Intercalary femur	Arthrodesis implant	Distal femur	Proximal tibia
All patients	114	53.4 (7.1–88.1)	55 (48.2)	116	15	1	1	3	5	30	3	4	2	33	19
Diagnosis															
Primary bone sarcoma	50	42.9 (7.1–86.0)	25 (50)	52	5	1	0	0	5	6	3	1	1	17	13
Soft tissue sarcoma	11	42.9 (16.4–73.8)	8 (80)	11	1	0	0	0	0	5	0	0	0	3	2
Benign bone tumour	6	58.0 (33.2–71.5)	3 (50)	6	1	0	0	0	0	1	0	0	1	2	1
Haematologic cancer	3	70.1 (63.6–80.1)	1 (25)	3	0	0	0	0	0	0	0	0	0	3	0
Metastasis from other cancer	38	66.5 (22.2–86.9)	16 (42)	38	7	0	1	3	0	17	0	3	0	4	3
Trauma or revision of other prosthesis	6	61.6 (29.5–88.1)	2 (33)	6	1	0	0	0	0	1	0	0	0	4	0

**Fig. 1:**
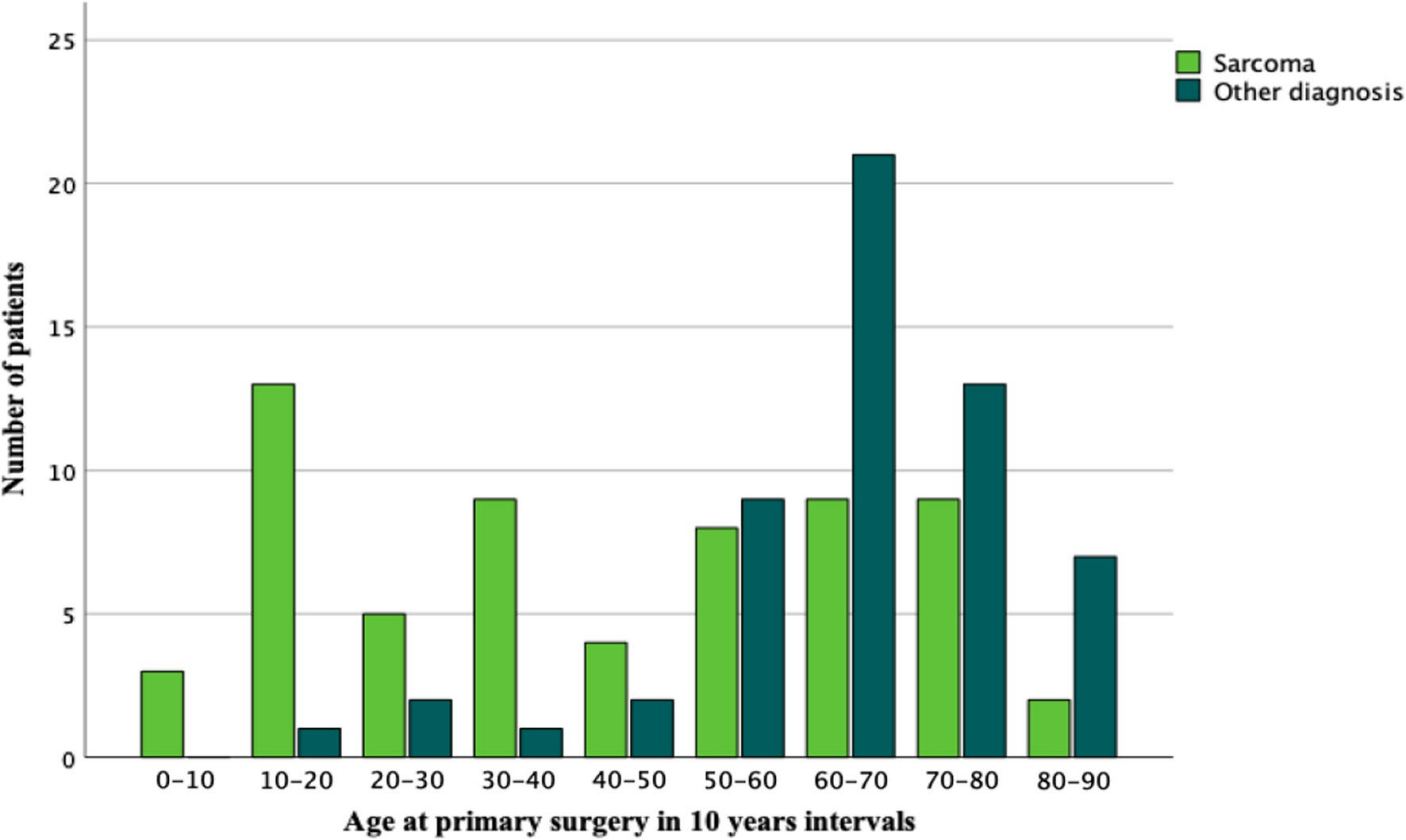
Age at primary surgery for sarcoma and other diagnosis as indication for surgery

Two peaks were identified in the age distribution—one in the adolescent-young adult age and one at around 70 years of age. The indication for surgery differed between the two age groups. The peak with the younger age group represented patients mostly diagnosed with sarcoma, and the peak with the older age group contained patients with other diagnoses (Fig. 1).

### Indication for mega prosthesis surgery

The predominant indication for primary surgery with a mega prosthesis was sarcoma of either bone or soft tissue origin (53.5% of the patients). Other malignancies (metastatic disease or haematologic malignancy) were the second most common indication (36.0%).

Patients operated due to a primary sarcoma had lower mean age (42.9 years) than patients operated due to another diagnosis (64.1 years).

Six of the patients had a mega prosthesis reconstruction due to benign bone tumour, i.e. giant cell tumour or pigmented villonodular synovitis (PVNS) with significant destruction of the affected joint.

**Fig. 2:**
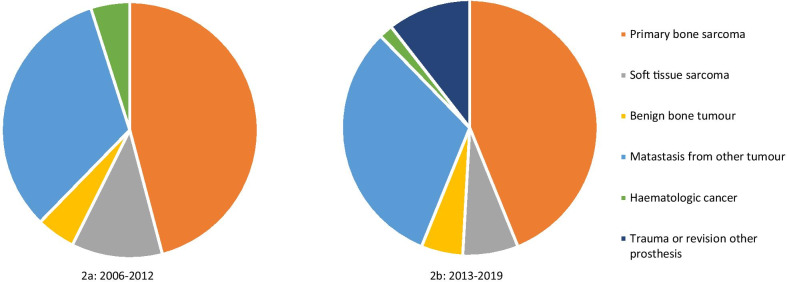
Indication for surgery first and second half of the study period

During the second half of the study period (2004–2019), mega prostheses were introduced as a surgical treatment option for selected trauma patients and for revisions of other endoprostheses; 10% of the patients in this study period received a mega prosthesis for these new indications. The distribution among the other diagnoses otherwise remained the similar during both halves of the study period (Fig. 2).

The distribution of patients according to diagnosis and anatomical location is presented in Table 1. In total six patients were provided with a MUTARS Expand™ prosthesis, one total femur and five distal femur prostheses.

### Reoperations and implant survival

In total 51 prostheses (44%) did not need any additional surgery after the primary operation, while 65 prostheses (56%) were subject to at least one reoperation. The number of additional surgeries in each patient differed largely between individuals. One patient underwent 51 procedures. Since 2011 no prosthesis was reoperated more than nine times in total.

**Fig. 3:**
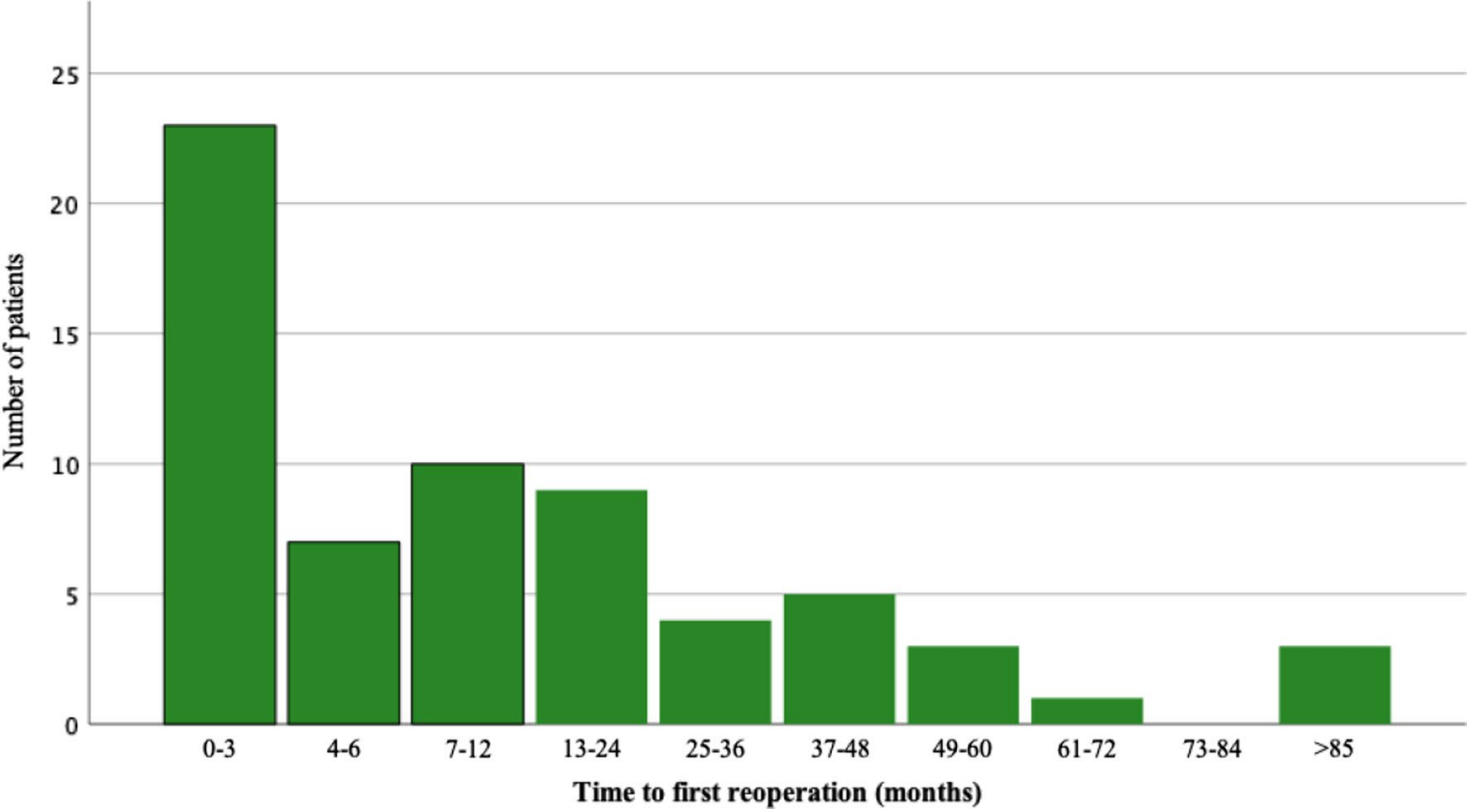
Time from primary surgery to first reoperation for any indication

The risk for a reoperation was highest during the first year after the primary surgery and after 2 years the risk decreased (Fig. 3). Indication for reoperations varied depending on length of time after the primary surgery was performed. Within the first year after primary surgery, infections and soft tissue failure were the dominant indications, while later it was mechanical failure and aseptic loosening (Table 2).

**Table 2:** Time to first reoperation for different indications

Indication for reoperation	Time from primary surgery to first reoperation (months)									Total
	0–3	4–6	7–12	13–24	25–36	37–48	49–60	61–72	> 85	
Soft tissue failure	8	2	4	1	0	0	0	0	0	15
Aseptic loosening	0	0	0	4	1	0	0	0	0	5
Mechanical complications	3	0	0	0	2	0	3	0	1	9
Infection	11	4	5	1	1	2	0	0	1	25
Tumour relapse/progression	0	1	0	2	0	1	0	1	1	6
Growth (children)	0	0	0	0	0	1	0	0	0	1
	22	7	9	8	4	4	3	1	3	61

Of the 65 patients who underwent reoperation for any of the five types of complications according to the Henderson classification [13], most patients had one (38 patients) or two (10 patients) reoperations. The most common cause for reoperation was infection (69.8% of all reoperations), followed by soft tissue failure (18.9%).

Reoperations included revision of the prosthesis, for any kind of complication, in 31 of the prostheses*.*

Infection was observed in 35 prosthesis, and as a result, some type of reoperation was required. Among these reoperations 19 included revision, ten in the form of a debridement and implant retention (DAIR) procedure and nine in the form of a two-stage procedure where the primary prosthesis was replaced with an antibiotic-containing bone cement spacer which was subsequently replaced with a secondary prosthesis, as described by Grimer et al. [15, 16]. The remaining 16 of 35 prostheses were surgically treated with debridement of the prosthesis without changing any components. At the end of the study period, 19 patients who had had an deep prosthesis infection at some time during the study period had no remaining signs of infection, four had signs of persistent infection and were planned to be treated with lifelong antibiotic suppressive therapy, two underwent an arthrodesis, and 10 had undergone amputation (Table 4).

**Fig. 4:**
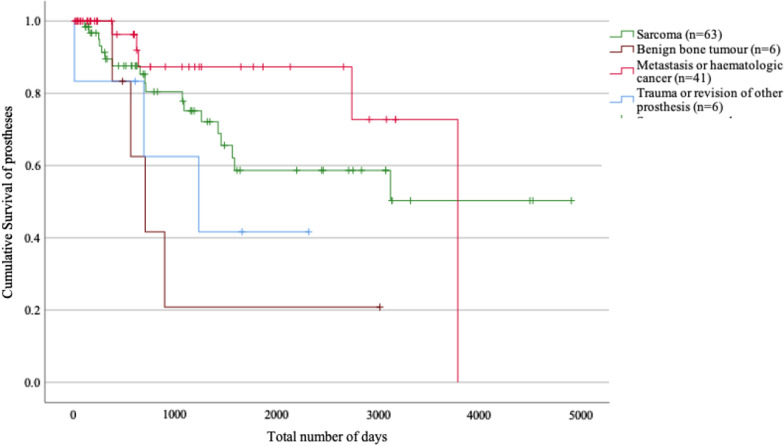
Survival of prostheses. Revision, extraction of prosthesis or amputation due to complication was set as event of interest. Patients who died during study period, were amputated or were revised due to tumour progression were censored

Figure 4 shows a Kaplan–Meier analysis of the survival of the implants where revision, extraction of the prosthesis or amputation due to complication was set as endpoints. Patients who died or underwent an amputation or revision due to tumour progression were censored.

Mechanical failure almost exclusively appeared within and/or around the knee joint and the most common failure was related to breakage of the PEEK™ locking mechanism. The mechanical problems for patients with proximal femur replacements were exclusively related to dislocations of the femoral head in the hip. Prosthetic replacement in the pelvis (LUMIC™) and in the proximal tibia was associated with the highest risk for reoperations (four out of five and 14 out of 19, respectively). The most common cause for reoperations of the prosthesis in the pelvis and proximal tibia was infection (60% and 26%, respectively) (Table 3).

**Table 3:** Number of prosthesis that underwent secondary surgery according to anatomical site and reason for first secondary surgery

	Total number of implants	Soft tissue failure	Aseptic loosening	Mechanical failure	Infection	Tumour relapse/progression	Total (%)
Site							
Proximal humerus	15	1	1	1	3	1	7 (47)
Total humerus	1	0	0	0	0	0	0 (0)
Diaphyseal humerus	1	0	1	0	0	0	1 (100)
Distal humerus	3	0	0	1	1	0	2 (67)
Pelvis	5	0	0	0	3	1	4 (80)
Proximal femur	30	4	0	3	4	2	13 (43)
Total femur	3	0	0	1	0	1	2 (67)
Arthrodesis implant	2	0	0	0	0	0	0 (0)
Diaphyseal femur	4	0	1	0	0	0	1 (25)
Distal femur	33	4	2	5	8	2	21 (61)
Proximal tibia	19	6	0	1	6	1	14 (68)
Total	116	15	5	12	25	8	65

### Amputation

Fifteen patients (12.9%) underwent amputation during the study period. Five were amputated due to tumour progression, among them four sarcoma and one metastasis from other malignancies, and ten were amputated due to periprosthetic infections. No other complications than tumour progression or infection led to amputation.

Periprosthetic infection resulted in a risk for amputation, especially when infection occurred in close relation to the knee joint. Altogether most of the infected implants in the distal femur (nine of twelve) and the proximal tibia (four of seven) required explantation or amputation to cure the infection (Table 4).

**Table 4:** Still implanted infected prosthesis and number of amputations due to infection for different surgical sites

	Total no. of prostheses	Infection no (%)	Rev. due to infection %	Infected prosthesis still impl.	Amputation due to infection
Proximal humerus	15	4 (27)	2 (13)	3	0
Total humerus	1	0 (0)	0 (0)	0	0
Diaphyseal humerus	1	0 (0)	0 (0)	1	0
Distal humerus	3	1 (33)	0 (0)	1	0
Pelvis	5	3 (60)	0 (0)	3	1
Proximal femur	30	7 (23)	4 (13)	3	1
Total femur	3	0 (0)	0 (0)	0	0
Arthrodesis implant	2	0 (0)	0 (0)	0	0
Diaphyseal femur	4	0 (0)	0 (0)	0	0
Distal femur	33	12 (38)	8 (24)	3	6
Proximal tibia	19	8 (42)	5 (26)	3	2
Total	116	35 (32)	19 (17)	17	10

The risk for a prosthetic infection was significantly higher in the group of patients diagnosed with sarcoma, compared with all other indications for surgery, regardless of surgical site (*p* = 0.004) (Table 5). A regression analysis including chemotherapy treatment, surgical site and resection length did not show any increased risk for infection or other complications with any of the tested parameters in this material.

**Table 5:** Risk for infection in relation to indication for surgery

	Infection *n* (%)		Reoperation *n* (%)		Total *n* (%)
	Yes	No	Yes	No	
Sarcoma	25 (41%)	36 (59%)	40 (66%)	21 (34%)	61 (52%)
Other diagnosis	10 (18%)	45 (82%)	25 (45%)	30 (55%)	55 (48%)
Total	35 (30%)	81 (70%)	65 (56%)	51 (44%)	116 (100%)

“Sarcoma” includes both bone and soft tissue sarcomas

At the end of the follow-up period for each patient, or at time of death, 98 patients (83%) still had a functional prosthesis in place, either a primary or revised one.

## Discussion

The most important finding of the current study is that despite a relatively large proportion of patients treated with a mega prosthesis requiring reoperation after the initial operation, most patients preserved a functioning limb with the use of a mega prosthesis implant. The reasons behind the high risk of complications leading to reoperations associated with mega prosthesis surgery are not completely determined. One can assume that large wound exposures and resections of the surrounding soft tissue, prolonged surgical time and the fact that the patients often are treated with chemotherapy and/or radiotherapy in near proximity to the operation might increase the risks [17]. Since the multivariate regression analysis in the current study showed no other significant risk factor for infection than sarcoma, it is difficult to predict the outcome or risk for complications for a certain patient.

The most severe complication, as in all surgery with implants, is infection. As shown in previous studies, the infection rate observed in the current study is much higher than after conventional endoprosthesis surgery [9]. High infection rate after surgery with mega prostheses has been described in several earlier studies. Bus et al., Capanna et al., Morii et al. and Fujiwara et al. showed infection rates of 12–17% [6, 17–19]. It is hard to explain why the infection rate in the current study is slightly higher than in these previous studies. One explanation might be different definitions of infected prostheses. Another interesting difference between the results of the current study and previous studies is that in the current study, the frequency of aseptic loosening is lower than in earlier studies. One might speculate that some of the prostheses diagnosed with aseptic loosening in previous studies may have been undiagnosed infections.

The current study shows the difficulties in treating an infection in a mega prosthesis successfully. For patients with an infection involving their mega prosthesis, half required extraction of the prosthesis and one of four resulted in amputation. This entails extensive suffering for the patients, not only because of the repeated surgery but also due to long antibiotic treatment and many days admitted into the hospital. In the current study, 70% of reoperations were due to postoperative infections. The total number of reoperations has not earlier been described in the manner presented herein, which makes it challenging to compare our numbers with previous studies. The high number of reoperations observed in this study is to an extent caused by difficulties in treating the infection. It would be of great benefit to identify the optimal treatment for infected mega prostheses and establish a routine, as is done for infections of conventional prostheses [20]. The relatively small number and diversity of patients, however, makes it difficult to perform a randomized controlled trial (RCT) comparing effect of different treatments for infection in this group of patients. In absence of RCTs, observational cohort studies like the current study can add important knowledge to the field.

A reconstruction with a mega prosthesis enables a patient to regain walking ability with full weight-bearing in close proximity to the surgery for tumour, fracture or endoprosthetic revision [1]. The impression from clinical practice is that a reconstruction using mega prostheses especially in the group of older patients with sometimes a high comorbidity improves the quality of life. However, this remains to be elucidated in future quality of life studies. Earlier studies have mainly focused on outcome for sarcoma patients, who tend to be young patients with high demands on function and often with strong medical anti-tumour treatments [18, 19].

One interesting observation in the current study is the shift over time in the indications for the use of a mega prosthesis. The use of mega prostheses for diagnoses other than primary bone tumours has been described in a previous review as well [5]. The indications have been expanded with good results and have thereby changed from merely a treatment option for primary bone tumours, to also become an alternative for complicated trauma, metastatic disease and revision of conventional prostheses. The findings from the current study demonstrate the benefit of mega prostheses also for patients outside the group of primary bone tumours. The lower rate of postoperative infection for patients treated because of indications other than sarcoma, might not only be caused by the absence of a tumour disease and/or tumour medical treatment, but may partially be due to careful selection of patients suitable for this type of surgery. The Kaplan–Meier analysis in the current study showed that the use of mega prostheses was a reliable option for patients with metastatic disease since most of them did not undergo reoperation and could keep their primary mega prosthesis for the rest of their lives. If surgery with a mega prosthesis in patients subjected to palliative care can help the patients to retain their walking capability, and reduce their pain, a mega prosthesis could be considered a good method, even if their remaining expected lifetime is short [21].

One could expect a better implant survival in the non-malignant group than for sarcoma patients, as the patients suitable for reconstruction with a mega prosthesis are carefully selected and does not undergo any anti-tumoural treatment. The seemingly worse survival of implants for benign tumours or fracture/endoprosthetic revision shown in this study therefor was surprising. In the fracture/endoprosthetic revision group one patient had an aseptic loosening of the implant, one suffered an infection, and one got a fracture of the femoral stem. Due to the small number of patients in this specific subgroup these complications had a large impact on the result. Until confirmed in larger cohorts the results in this group of patients need to be interpreted with caution.

Not all surgeries required after the primary operation can be attributed due to complication but sometimes should be considered more as “service procedures” due to wear or growing of a young patient [22]. In the current study, it was found that the highest risk for reoperation for sarcoma patients is during the first 2 years after the primary surgery and that after 5 years there was a low risk for reoperations. One reasonable explanation for this might be the consequences after anti-tumour treatment often given for several months after the surgery. Further studies aiming to find methods to protect patients from complications the first years after the primary surgery are warranted.

### Strengths and limitations

Limitations within the current study are the single-centre design and the relatively small number of patients. However, this also enabled the relatively long follow-up and very few patients were lost to follow-up, since all patients but one remained for care at the centre with long-term data available, which is a strength of the study.

Another limitation is that this study cannot determine the final function and whether the patients suffer from pain postoperatively because no functional, quality of life or pain score was included in the study design. The use of mega prostheses is still limited to highly specialised units and for specific patients, why it can be difficult to achieve large cohorts and every case has a big impact on the overall outcome in cohort studies. This shows, as earlier described [23], the need for prospective databases used by multiple centres/nations for structured collection of data regarding complicating events after reconstructions with mega prostheses.

## Conclusion

The study reveals a total reoperation rate of 56% after reconstructive surgery using mega prostheses. Despite the high reoperation rate, at the end of the study period, 83% of the patients still had a functioning prosthesis. Therefore, mega prosthesis can be considered a reliable method for reconstruction of large bone defects, not only for sarcoma patients but also, and maybe even with better results, for other diagnoses entailing bone defects.

## Data Availability

The datasets used and analyzed during the current study are available from the corresponding author on reasonable request.

## Abbreviations

PVNS: Pigmented villonodular synovitis
MUTARS™: Modular Universal Tumour and Revision System
DAIR: Debridement, antibiotics and implant retention
STROBE: Strengthening the Reporting of OBservational studies
